# Spinal impostor: Metastatic cervical paraganglioma presenting with paraparesis, a case report

**DOI:** 10.1016/j.ijscr.2025.110821

**Published:** 2025-01-08

**Authors:** Musa Machibya, Abduel Kitua, Jackline Gabone, Nuru Saleh, Caroline Ngimba, Mugisha Clement

**Affiliations:** a Department of Surgery, The Aga Khan University, P. O Box 38129, Dar Es Salaam, Tanzania; b Neurosurgery Section, Department of Surgery, The Aga Khan Hospital, P. O Box 2289, Dar Es Salaam, Tanzania; c Department of Radiology, The Aga Khan Hospital, P. O Box 2289, Dar Es Salaam, Tanzania; d Department of Oncology, The Aga Khan Hospital, P. O Box 2289, Dar Es Salaam, Tanzania; e Department of Pathology, The Aga Khan Hospital, P. O Box 2289, Dar Es Salaam, Tanzania

**Keywords:** Spinal paraganglioma, Case report, Diagnostic uncertainty, Neoplastic cord compression, Carotid body tumor

## Abstract

**Introduction and importance:**

Paragangliomas are rare neuroendocrine tumors, typically arising from extra-adrenal chromaffin cells. Primary intra-spinal paragangliomas are uncommon, and metastatic spinal paragangliomas without paraneoplastic symptoms are even rarer. This case highlights the diagnostic challenges posed by such rare tumors.

**Case presentation:**

A 28-year-old male soldier from the Comoros Islands presented with a neck mass, initially suspected to be Hodgkin's lymphoma based on imaging. Biopsy of two cervical nodes revealed reactive lymphadenopathy. Later, he developed progressive lower limb weakness and numbness, prompting further investigation. Imaging showed an extradural spinal tumor at T6 with cord compression. Laminectomy and tumor excision relieved compression, revealing a highly vascularized tumor. Histopathology and immunohistochemistry confirmed a paraganglioma, which was consistent with the metastatic nature confirmed by a repeat biopsy of the neck mass.

**Clinical discussion:**

Metastatic spinal paragangliomas are rare and challenging to diagnose, especially without paraneoplastic symptoms. This case underscores the importance of thorough histopathological evaluation when spinal lesions and neck masses present with unusual features and highlights the need for a multidisciplinary approach.

**Conclusion:**

This case emphasizes the diagnostic difficulty of metastatic spinal paragangliomas, particularly when they mimic more common conditions like Hodgkin's lymphoma. It stresses the importance of considering rare differential diagnoses and a collaborative approach to managing such cases.

## Highlights

• Rare presentation: Cervical Paraganglioma with spine metastasis
• Diagnostic challenge: Needing imaging and multiple biopsies for histopathological diagnosis
• Management dilemma: Surgery was required for diagnosis and treatment due to spine metastasis and unclear malignancy

## 1 Introduction

Paragangliomas are rare neuroendocrine neoplasms that originate from extra-adrenal chromaffin cells of the autonomic nervous system. First identified by Alfred Kohn in 1903, as the lesions originating from neural crest-derived cells which exhibit extensive vascularization and are intimately connected with tissues involved in autonomic functions [1].

These tumors exhibit indolent growth and often present with systemic manifestations due to catecholamine release, as well as symptoms resembling carcinoid syndrome [2]. The World Health Organization classifies paragangliomas as grade I tumors [3].

The combined incidence of paragangliomas and pheochromocytomas is approximately 0.7 to 1.0 per 100,000 person per years, with a slight male predominance, and they are most diagnosed in individuals aged 30 to 50 years [4].

Central nervous system (CNS) paragangliomas are rare, with over 90 % occurring as carotid body and glomus jugular tumors [5]. Spinal paragangliomas are exceedingly rare, predominantly affecting the lumbar region, with the thoracic region being less frequently involved [6,7].

Paragangliomas can mimic other conditions such as lymphomas, schwannomas, thyroid tumors, or vascular anomalies which can present a challenge in making a diagnosis due to overlapping clinical features. On imaging, paragangliomas often show increased vascularity and may involve the spine in metastatic cases. Accurate diagnosis requires careful clinical assessment, radiologic imaging, and histopathological analysis [18].

These tumors are typically benign, with a 5-year survival rate of approximately 90 %. However, the malignant transformation rate ranges from 2.4 % to 14 %. The prognosis is favorable for localized tumors, whereas metastatic disease carries a poorer prognosis, with a 5-year survival rate of 40–60 %. The local recurrence rate is 2.2 % following total excision and ranges from 5.4 % to 10.5 % following subtotal excision [2,8,9]. When metastatic, osseous metastases are most common with about a third of the cases presenting as synchronous metastases [10]. The most frequent skeletal metastases sites for malignant paraganglioma and pheochromocytoma are spine followed by sacrum and pelvis with about 25 % of these patients presenting with spinal cord compression and neurological deficits [10].

Treatment for metastatic cervical paraganglioma causing neurological deficit typically involves surgical resection, radiation therapy for inoperable metastases, and systemic therapies like chemotherapy or targeted treatments (e.g., tyrosine kinase inhibitors), followed by post-operative rehabilitation focusing on physical and occupational therapy to restore function, manage pain, and support psychological well-being [19].

The aim of this report was to report a rare case of cervical carotid paraganglioma with solitary thoracic spine metastasis and to conduct a comprehensive review of the related literature, highlighting diagnostic challenges and management dilemma of these accounts.

This case report has been reported in line with Surgical Case Report guidelines (SCARE) [17].

## 2 Case presentation

We present this case of a 28-year-old gentleman, with a previously documented neck swelling suspicious for Hodgkin's Lymphoma and negative histopathological evaluation of initial excisional cervical adenopathy biopsies and was then lost to follow up. He later presented with progressive bilateral lower limb weakness over an 8-month duration. This weakness gradually worsened, leading to an inability to walk or perform daily activities. Additionally, he experienced a gradual onset of loss of sensation from the umbilicus downward to the lower limbs. Notably, there was no history of trauma, falls, incontinence and changes in bowel or bladder habits. Furthermore, weight loss and constitutional symptoms were absent. Preceding these symptoms, was gradual-onset low back pain exacerbated by activity and relieved by analgesia and rest. He also reported intermittent episodes of dizziness without associated tinnitus, vertigo, or loss of consciousness.

**Fig. 1:**
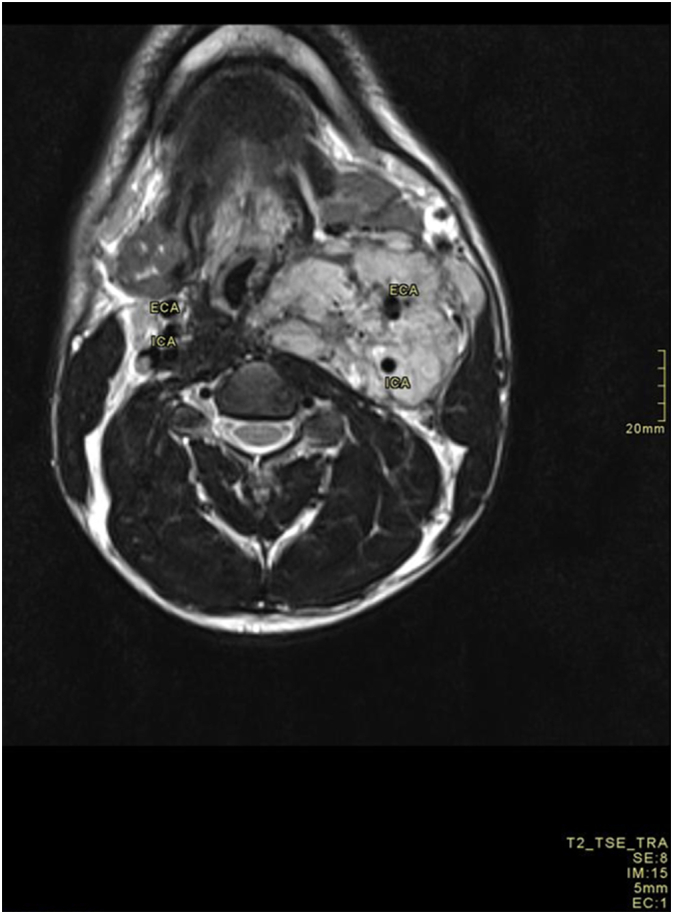
Cervical MRI images, T1 FS with contrast Axial demonstrating the vessel encasing left carotid paraganglioma.

The patient had a previous hospital admission in December 2021, 2 years prior to index presentation, for a painless anterolateral neck swelling associated with difficulty breathing and swallowing. Examination revealed a firm, fixed, non-tender, pulsatile mass measuring 12 by 5 cm, with multiple lymph nodes palpable along its course. Initial investigations raised suspicion for lymphoma, with concerns for tuberculosis adenitis. MRI of the neck demonstrated an enhancing left neck mass encasing the great vessel, compressing the trachea (Fig. 1), suggestive of a carotid body tumor (Shamblin group III), with a differential diagnosis including Hodgkin's Lymphoma. Further evaluation with CT chest showed multiple small left axillary lymph nodes raising suspicion for lymphoma. However, after two consecutive excisional biopsies of the cervical lymph nodes, histopathological assessment and immunohistochemistry were inconsistent with Lymphoma and conclusion, in collaboration with re-evaluation at an external pathology laboratory, revealed reactive lymphadenitis, precluding definitive diagnosis and the patient was lost to follow up until index presentation.

The patient, married with one child and working as a soldier in the Comoro Islands, had a history of regular alcohol consumption and chronic smoking, although he ceased smoking upon the onset of his illness. Family and social support were deemed adequate during his illness.

**Fig. 2:**
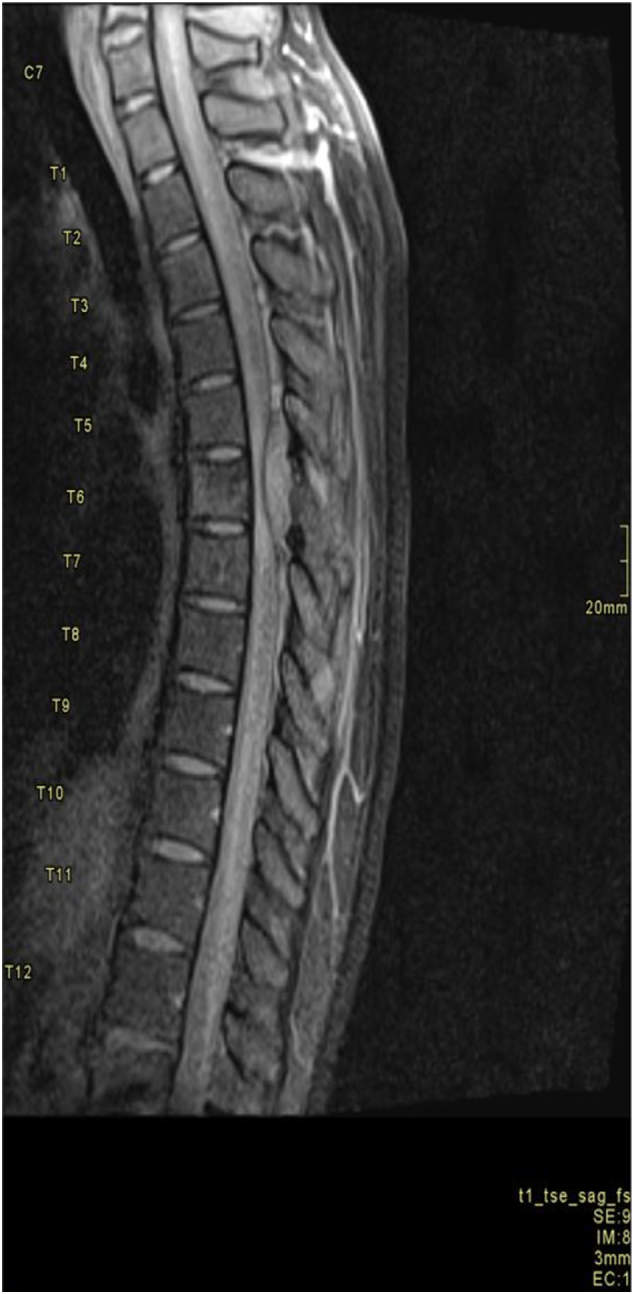
Thoracic spine MRI with contrast, T1 FS Sagittal demonstrating T6 spinal veterbra metastasis with cord compression.

On physical examination, he was alert, afebrile, and mild pallor, with stable vital signs. Neurological assessment revealed intact cognition, memory, and normal upper limb motor function. However, there was reduced lower limb muscle bulk, increased tone, and grade 3/5 power bilaterally, with hyperreflexia noted. Laboratory investigations demonstrated leukocytosis, anemia, and elevated slightly C-reactive protein levels. MRI of the spine revealed an extradural spine lesion extending to the vertebra body and transverse process at the T6 level causing significant cord compression (Fig. 2).

Given the radiological findings and neurological status, the patient underwent T6 laminectomy during which a firm, highly vascularized extradural tumor was encountered, and maximal safe spinal tumor excision was performed maintaining an intact dura (Fig. 6). Intraoperatively, significant blood loss necessitated transfusion of 4 units of blood. Biopsy samples revealed an extradural paraganglioma with differential considerations for metastatic clear cell carcinoma. Immunohistochemistry confirmed the diagnosis, highlighting positive staining for chromogranin, pancytokeratin, CD56, Vimentin, S100 and Synaptophysin (Fig. 4, Fig. 5).

**Fig. 3:**
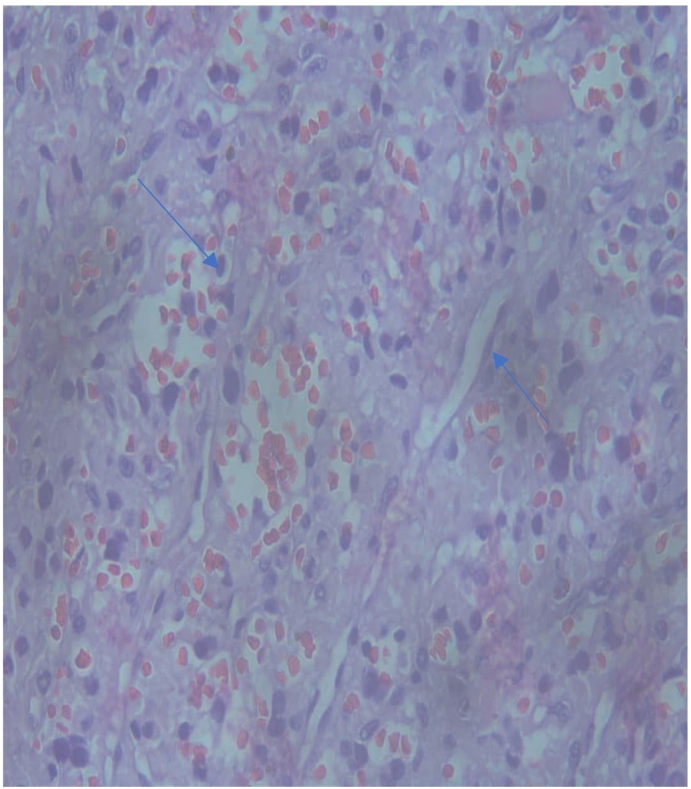
H & E neck mass (Carotid body) showing Tumor in nests and sheets of uniform atypical cells separated by delicate fibrovascular cores (Zellballen Pattern-blue arrow). (For interpretation of the references to colour in this figure legend, the reader is referred to the web version of this article.)

Re-evaluation of the neck mass was conducted by obtaining a third incisional biopsy on the neck lesion whereby massive intraoperative blood loss was encountered but subsequently achieved hemostasis. The histopathological examination of the sample revealed a paraganglioma, which was further confirmed by immunohistochemistry and confirming the metastatic nature of the spine lesion from this primary cervical carotid paraganglioma (Fig. 3).

**Fig. 4:**
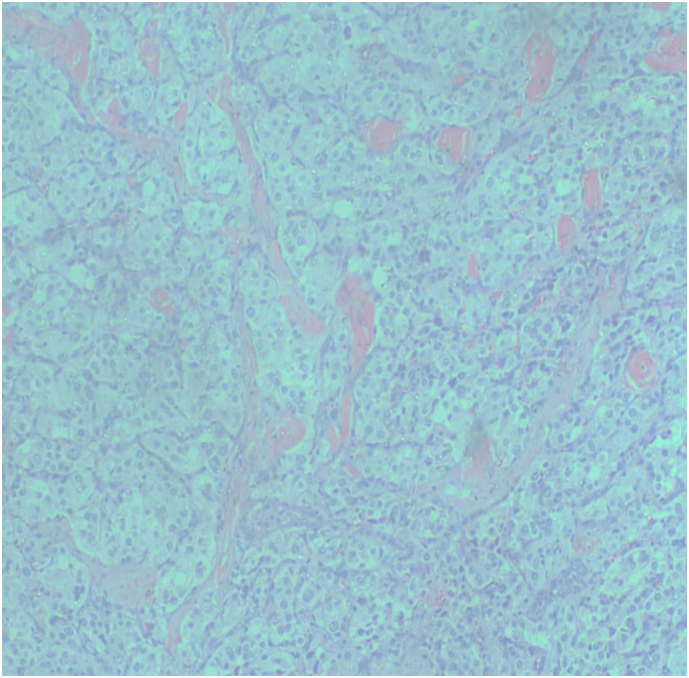
H&E Spinal metastasis; (low power)-Sections show a tumor in nested/zellballen pattern separated by delicate fibrovascular cores.

**Fig. 5:**
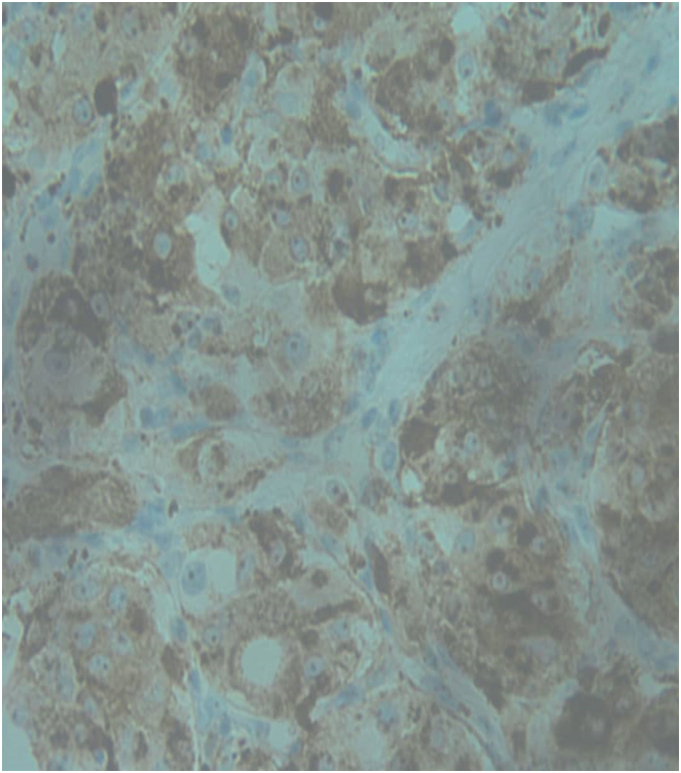
Chromogranin Immunohistochemistry staining with high power showing strong and diffuse staining of tumor cells for chromogranin.

**Fig. 6:**
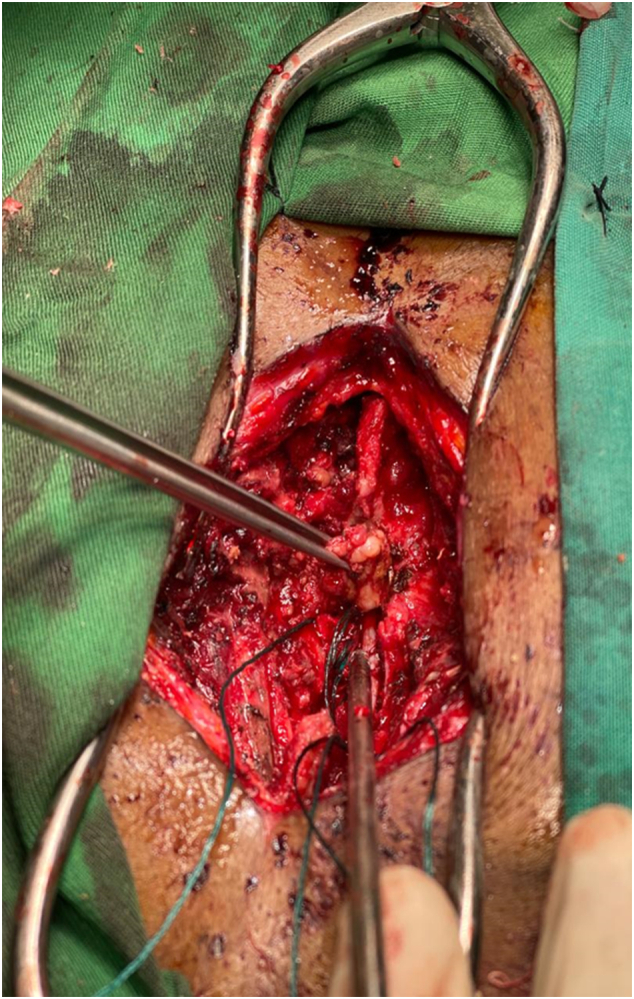
Intra-operative images of spine metastasis resection.

Further management involved postoperative monitoring and extended follow-up. The patient demonstrated significant improvement in lower extremity strength and sensation postoperatively, achieving independent ambulation and was discharged home on the fourth postoperative day. Our hospital's multidisciplinary tumor board reviewed the case, and the patient was recommended to receive radiotherapy and chemotherapy for management of metastatic spinal paraganglioma, with the primary tumor located in the carotid body. The patient, however, opted to pursue further treatment abroad.

We are in close coordination with the medical team abroad regarding the long-term follow-up of this patient, who is currently undergoing targeted radiotherapy and chemotherapy for metastatic spinal paraganglioma. Regular follow-up, including serial imaging, is being conducted to monitor for any recurrence or progression of the disease, and assessments for potential endocrine changes related to the tumor's neuroendocrine activity have so far been unremarkable. Additionally, the patient has shown no neurological deficits, and his functional recovery is being supported through physical therapy. He is also receiving psychosocial care to address the emotional aspects of his condition.

## 3 Discussion

Spinal paragangliomas are rare, and their origin is uncertain due to the absence of paraganglia in this region (whether from sympathetic autonomic medullary nerves or heterotopic cells). They are most frequently located in the intradural extramedullary compartment of the lumbosacral tract [11]. Evidence indicates that approximately 80 % to 90 % of paraganglioma cases originate in the carotid body or jugular vein, though they can also occur in other locations, such as the thoracic spine, where they are rare. When these tumors do manifest primarily in the spine, they are predominantly intradural and situated in the cauda equina [[5], [6], [7]]. Contrary to our case, the tumor was at thoracic spine T6 level and extradural in keeping with the possibility of a secondary metastasis. Furthermore, in 2022, during his prior evaluation for lymphoma, there was no evidence of spine involvement noted on the contrast enhanced Computed tomography of the chest acquired in extension for further evaluation of the cervical mass thus increasing likelihood of secondary involvement.

According to John et al. cited by Gelabert-González, Constantini et al., and Cruz Ortiz et al., several cases of thoracic spine paraganglioma were reported, all presenting with spinal cord compression. These cases share similarities with our patient, where the tumor's onset was insidious over several months. The significant spinal cord compression observed in our case suggests that the tumor had been developing for an extended period, likely growing slowly until the time of presentation, akin to the previously reported cases [1,12,13].

The clinical manifestations of intraspinal paragangliomas can vary depending on the tumor's location, primarily involving back pain and spinal cord compression symptoms presenting as focal neurological weakness and sensory disturbances as was seen in our case. The similar findings and clinical manifestation were reported by the study which was done by Mishra et al. [21]. Additional symptoms such as hypertension, heart palpitations, tremors, vomiting, and weight loss may occur due to elevated catecholamine levels in the blood or urine [2]. There was no evidence of paraneoplastic syndrome in our presented patient who was otherwise normotensive, and his urine was negative for elevated metanephrines.

As the initial diagnostic tool, MRI effectively displays well-demarcated masses, which appear hypo- to iso-intense on T1-weighted imaging (T1WI) and iso- to hyper-intense on T2-weighted imaging (T2WI). These masses exhibit homogeneous gadolinium enhancement, heterogeneous enhancement in tumor blood vessels, and cystic changes. Additionally, contrast-enhanced homogeneity on T1WI and the “salt and pepper” vascularization pattern on T2WI aid in identifying these tumors. The “salt and pepper” appearance, along with serpiginous flow voids and peripheral hypointense rims due to hemosiderin, further assist in diagnosing paragangliomas which is like our case and other published spine paraganglioma cases [14,15].

Paragangliomas are highly vascularized tumors, complicating complete surgical resection. Literature documents recurrence in cases where total resection is not achieved. The local recurrence rate is 2.2 % following total excision and ranges from 5.4 % to 10.5 % following subtotal excision [2,9]. In our case, despite the technical difficulties and significant hemorrhage encountered during the resection the highly vascularized tumor, maximal safe resection was achieved at the expense of incomplete resection of the T6 transverse process making the possibility of local spine recurrence likely.

The role of postoperative radiotherapy or chemotherapy in cases of paragangliomas is controversial. It is recommended for patients who do not undergo surgery as these treatments have limited efficacy and are typically used palliatively in cases of aggressive or multicentric paragangliomas, or when the patient's condition precludes surgery. Postsurgical radiotherapy is also employed in cases of recurrence or following the resection of metastases, however, its efficacy in paragangliomas, especially metastatic cases, remains controversial and it tend to have some side effects such as radiation-induced tissue damage, fatigue, and potential neurotoxicity which should be carefully considered in the management plan [16]. Similarly, chemotherapy and other systemic therapies, such as tyrosine kinase inhibitors, may be considered, particularly for patients with aggressive or multicentric disease. Yet, evidence supporting their effectiveness is limited, and these treatments are often used in select patients with high-risk features or when surgery alone is insufficient [20].

This case report on metastatic cervical paraganglioma presents valuable insights but has several limitations, such as being based on a single patient, which restricts the generalizability of the findings. The delayed diagnosis, misidentification as Hodgkin's lymphoma, and challenges with surgical resection due to blood loss further highlight the diagnostic and therapeutic complexities. Early and accurate diagnostic methods, including advanced imaging and histopathological evaluation, should be prioritized to minimize delays in diagnosis. Moreover, more comprehensive data on the effectiveness of various treatments, including surgery, radiotherapy, and chemotherapy, would help guide clinical decision-making. A multi-center, collaborative approach is recommended to gather more robust evidence, and attention to the psychosocial and quality-of-life aspects of patients with rare tumors is crucial for providing holistic care and improving long-term outcomes.

## 4 Conclusion

Spinal paragangliomas, though rare and generally benign, pose significant diagnostic challenges due to their clinical presentation and overlapping imaging features with other conditions. MRI plays a crucial role in identifying the tumor's location, vascularity, and the extent of involvement, including spinal cord compression. The characteristic “salt and pepper” appearance on T2-weighted images is a key diagnostic feature. However, definitive diagnosis requires histopathological and immunohistochemical confirmation, with markers like chromogranin and synaptophysin typically present. Metastatic paragangliomas are even rarer and share similar diagnostic difficulties, often becoming apparent only when neurological deficits occur. Treatment typically involves maximal surgical resection, with adjuvant radiotherapy being recommended for cases of recurrence or metastasis. The use of chemotherapy, radiotherapy, and other therapies like octreotide remains controversial, and these options are considered based on the individual patient's condition. Long-term follow-up is essential to monitor for recurrence and refine treatment strategies for this complex and rare condition.

## CRediT authorship contribution statement

1. Musa Machibya: Study conception, production of initial article, collection of data, final manuscript writing
2. Abduel Kitua: Study conception, revision of the article, proofreading, final manuscript writing
3. Nuru Saleh: Study conception, revision of the article, and proofreading
4. Jackline Gabone: Study conception, revision of the article, and proofreading
5. Caroline Ngimba: Study conception, revision of the article, and proofreading
6. Mugisha Clement: Study conception, revision of the article, proofreading, final manuscript writing

## Consent

The patient provided written informed consent for publication and for the inclusion of any related images. A copy of the written consent is available for review by the Editor-in-Chief of this journal on request.

## Ethical approval

Written informed consent was obtained from the patient for the anonymized information to be published in this article.

Our institution does not require ethical approval for reporting individual cases report and the case reports are exempted from ethical approval in our institution, the Aga Khan University Medical College, Tanzania (Dar es salaam). P.O. Box 38129, Dar es Salaam. T: (Office) +255 22 212 2740 | 212 2744.

## Guarantor

Dr. Musa Machibya

The Aga Khan University, Dar es salaam

Email Address; musa.machibya@scholar.aku.edu

## Research registration number

1. Name of the registry: N/A
2. Unique identifying number or registration ID: N/A
3. Hyperlink to your specific registration (must be publicly accessible and will be checked):

## Funding

The author(s) received no financial support for the research, authorship, and/or publication of this article.

## Declaration of competing interest

The author(s) declared no conflicts of interest regarding the research, authorship, and/or publication of this article.
